# Supplementary material for: Computer-aided discovery of a metal–organic framework with superior oxygen uptake
Source: Nat Commun. 2018 Apr 11;9:1378. doi: 10.1038/s41467-018-03892-8 (PMC5895810; doi:10.1038/s41467-018-03892-8)
Supplement: Supplementary file 1 — Supplementary Information [file 41467_2018_3892_MOESM1_ESM.docx]

# *Supporting Information for:*

**Computer-Aided Discovery of a Metal-Organic Framework with Superior Oxygen Uptake**

Peyman Z. Moghadam*^a,*^*, Timur Islamoglu*^b^*, Subhadip Goswami*^b^*, Jason Exley*^c^*, Marcus Fantham*^a^*, Clemens F. Kaminski*^a^*, Randall Q. Snurr*^d^*, Omar K. Farha*^b,*^*, and David Fairen-Jimenez*^a,*^*

^a^Department of Chemical Engineering & Biotechnology, University of Cambridge, Philippa Fawcett Drive, Cambridge CB3 0AS, UK

^b^Department of Chemistry, Northwestern University, Evanston, Illinois 60208, United States

^c^Particulate Systems, Micromeritics Instrument Corp. 4356 Communications Drive, Norcross, Georgia 30093, United States

^d^Department of Chemical and Biological Engineering, Northwestern University, Evanston, Illinois 60208, United States

*e-mail: pzm20@cam.ac.uk, o-farha@northwestern.edu, df334@cam.ac.uk

**Supplementary Figure 1.** Relationship between the oxygen deliverable capacity and oxygen uptake at 140 bar for 2,932 MOF structures at 298 K**.** The data points are color coded and sized by heat of adsorption and void fraction, respectively. Each point in the graph represents a different structure.

| **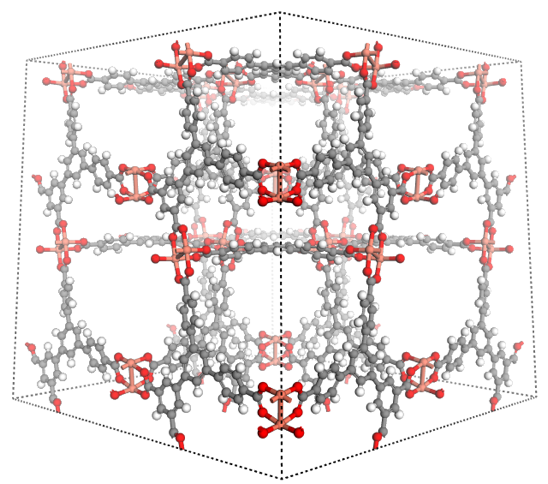** | **CCDC ref code:** ANUGIA (UMCM-152)  **LCD:** 13.8 Å  **PLD:** 6.8 Å  **Surface area:** 3760 m**^2^/g**  **Density:** 0.57 g/cm^3^  **Void fraction:** 0.86  **Volumetric deliverable cap.:** 248.9 cm^3^(STP)/cm^3^  **Gravimetric deliverable cap.:** 19.6 mol/kg |
| --- | --- |

| **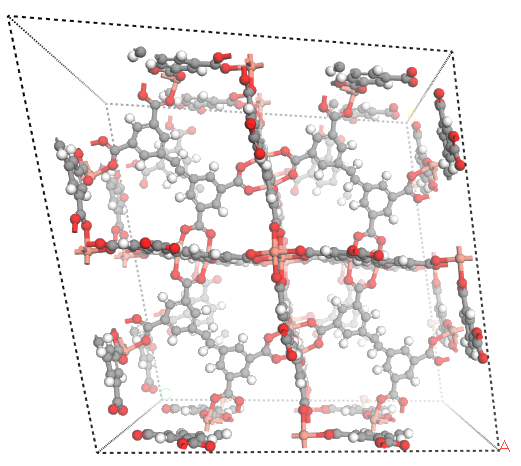** | **CCDC ref code:** MOCKAR  **LCD:** 10.8 Å  **PLD:** 6.7 Å  **Surface area:** 2948 m^2^/g  **Density:** 0.75 g/cm^3^  **Void fraction:** 0.83  **Volumetric deliverable cap.:** 243.4 cm^3^(STP)/cm^3^  **Gravimetric deliverable cap.:** 14.5 mol/kg |
| --- | --- |

| **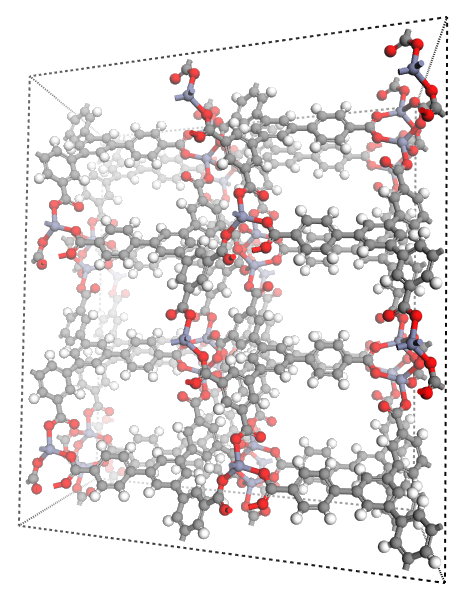** | **CCDC ref code:** ANUGUM  **LCD:** 8.2 Å  **PLD:** 7.0 Å  **Surface area:** 3444 m^2^/g  **Density:** 0.68 g/cm^3^  **Void fraction:** 0.81  **Volumetric deliverable cap.:** 241.3 cm^3^(STP)/cm^3^  **Gravimetric deliverable cap.:** 15.8 mol/kg |
| --- | --- |

**Supplementary Figure 2.** Top 10 crystal structures among 2,932 MOF materials with the highest volumetric oxygen deliverable capacity at 298 K. The geometric properties for each structure are shown on the right along with volumetric and gravimetric oxygen deliverable capacity at 140 bar storage and 5 bar release pressures.

| **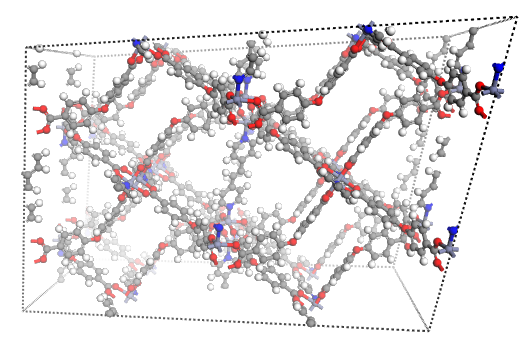** | **CCDC ref code:** DIDDOK  **LCD:** 9.6 Å  **PLD:** 8.2 Å  **Surface area:** 4639 m^2^/g  **Density:** 0.53 g/cm^3^  **Void fraction:** 0.83  **Volumetric deliverable cap.:** 240.2 cm^3^(STP)/cm^3^  **Gravimetric deliverable cap.:** 20.4 mol/kg |
| --- | --- |
| **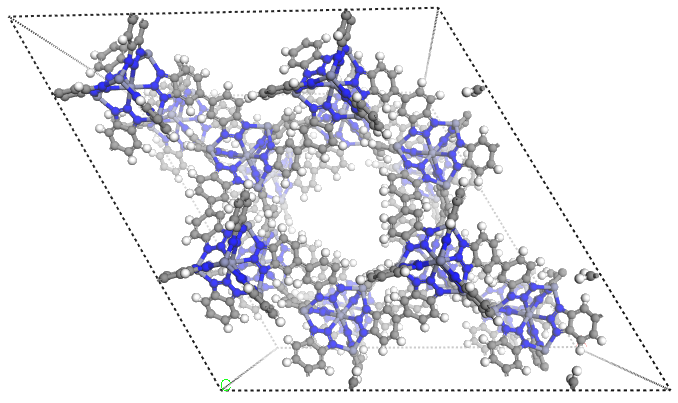** | **CCDC ref code:** BICDAU  **LCD:** 11.5 Å  **PLD:** 6.7 Å  **Surface area:** 3557 m^2^/g  **Density:** 0.65 g/cm^3^  **Void fraction:** 0.84  **Volumetric deliverable cap.:** 239.0 cm^3^(STP)/cm^3^  **Gravimetric deliverable cap.:** 16.3 mol/kg |

| **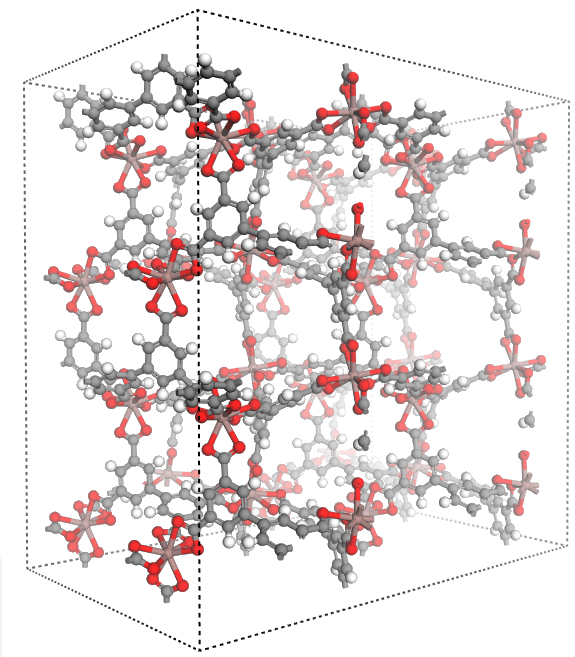** | **CCDC ref code:** HIHNUJ  **LCD:** 8.2 Å  **PLD:** 8.0 Å  **Surface area:** 2772 m^2^/g  **Density:** 0.81 g/cm^3^  **Void fraction:** 0.83  **Volumetric deliverable cap.:** 238.9 cm^3^(STP)/cm^3^  **Gravimetric deliverable cap.:** 13.1 mol/kg |
| --- | --- |

**Supplementary Figure 2:** Continued.

| **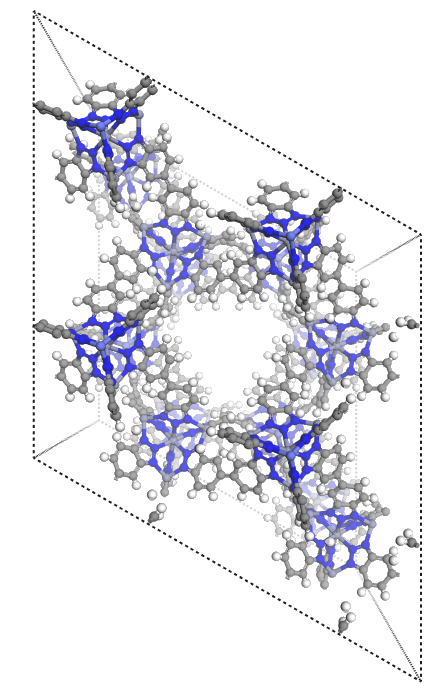** | **CCDC ref code:** HIGRIA  **LCD:** 11.3 Å  **PLD:** 6.9 Å  **Surface area:** 3477 m^2^/g  **Density:** 0.65 g/cm^3^  **Void fraction:** 0.84  **Volumetric deliverable cap.:** 238.7 cm^3^(STP)/cm^3^  **Gravimetric deliverable cap.:** 16.4 mol/kg |
| --- | --- |
| **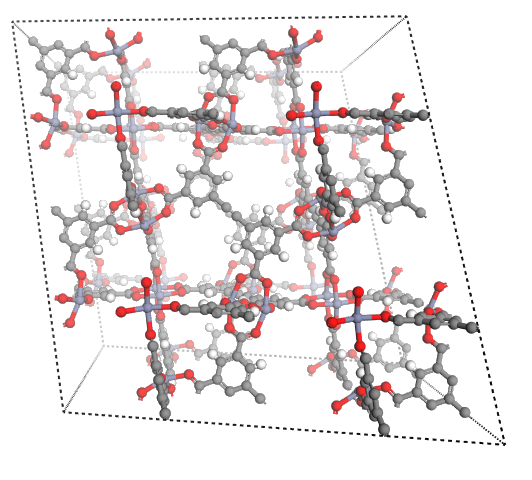** | **CCDC ref code:** KEFBEE  **LCD:** 11.1 Å  **PLD:** 7.0 Å  **Surface area:** 3087 m^2^/g  **Density:** 0.7 g/cm^3^  **Void fraction:** 0.82  **Volumetric deliverable cap.:** 238.0 cm^3^(STP)/cm^3^  **Gravimetric deliverable cap.:** 15.0 mol/kg |

| **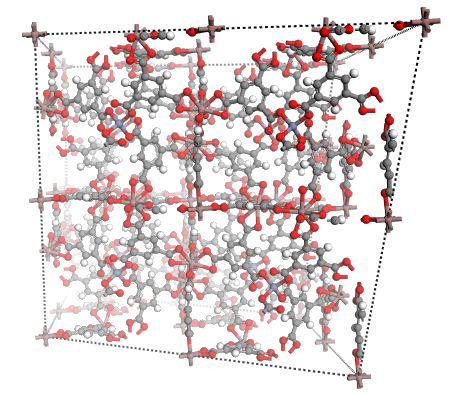** | **CCDC ref code:** WEBKOF  **LCD: 9.6** Å  **PLD:** 6.5 Å  **Surface area:** 2592 m^2^/g  **Density:** 0.85 g/cm^3^  **Void fraction:** 0.80  **Volumetric deliverable cap.:** 237.2 cm^3^(STP)/cm^3^  **Gravimetric deliverable cap.:** 12.4 mol/kg |
| --- | --- |

**Supplementary Figure 2:** Continued.

| **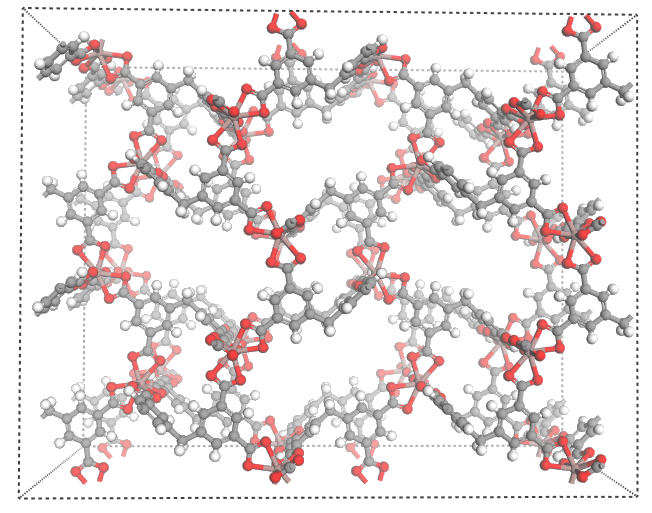** | **CCDC ref code:** ICALOP  **LCD: 7.5** Å  **PLD:** 6.0 Å  **Surface area:** 2908 m^2^/g  **Density:** 0.80 g/cm^3^  **Void fraction:** 0.82  **Volumetric deliverable cap.:** 236.2 cm^3^(STP)/cm^3^  **Gravimetric deliverable cap.:** 13.2 mol/kg |
| --- | --- |

**Supplementary Figure 2:** Continued.

**Supplementary Figure 3.** Pore size distribution (PSD) for UMCM-152.

**Supplementary Method 1**

**Molecular simulation details**

The simulated adsorption of O_2_ and N_2_ was investigated using grand canonical Monte Carlo (GCMC) simulations performed with the multi-purpose simulation package RASPA.^1^ The framework atoms were kept fixed at the crystallographic positions for all MOFs in the database.^2^ We used the standard Lennard-Jones (LJ) 12-6 plus Coulomb potential to model the interactions between fluid/fluid and fluid/framework atoms. The LJ parameters for the framework atoms were obtained from the UFF force field^3^ (also Dreiding^4^ for N_2_ adsorption), and the framework charges were taken from the work of Nazarian *et al*.^2^ O_2_^5^ and N_2_^6^ were modeled using the TraPPE potential with charges placed on each atom and at the center of mass. The Lorentz-Berthelot mixing rules were employed to calculate fluid/solid LJ parameters, and LJ interactions beyond 12.8 Å were neglected. The Ewald sum method was used to compute the electrostatic interactions. 10^4^ Monte Carlo cycles were performed, the first 50% of which were used for equilibration, and the remaining cycles were used to calculate the ensemble averages. To calculate the gas-phase fugacity, we used the Peng-Robinson equation of state.

**Supplementary Table 1.** Lennard-Jones parameters for UMCM-152, N_2_^6^ and O_2_^5^.

| Atom | **σ [Å]** | **ε/k [K]** |  | **σ [Å]** | **ε/k [K]** | **q [e]** |
| --- | --- | --- | --- | --- | --- | --- |
|  | **UFF** | |  | **Dreiding** | |  |
| C_MOF_ | 3.431 | 52.838 |  | 3.472 | 47.856 |  |
| O_MOF_ | 3.118 | 30.218 |  | 3.033 | 48.158 |  |
| H_MOF_ | 2.571 | 22.142 |  | 2.846 | 7.648 |  |
| Cu_MOF_ | 3.114 | 2.518 |  | – | – |  |
|  | **TraPPE** | |  |  |  |  |
| N_N_2_ | 3.310 | 36.000 |  |  |  | -0.482 |
| N_com | 0 | 0 |  |  |  | 0.964 |
| O_O_2_ | 3.020 | 49.000 |  |  |  | -0.113 |
| O_com | 0 | 0 |  |  |  | 0.226 |

**Supplementary Figure 4.** N_2_ adsorption isotherms at 77 K for UMCM-152, **a.** linear scale and **b.** semi-logarithmic scale. Simulated (closed black circles, Universal Force Field (UFF); open blue diamonds, Dreiding) and experimental (open red circles) data.

**Supplementary Figure 5.** Relationship between absolute volumetric oxygen adsorption and the largest cavity diameter (LCD) for 2,932 MOF structures at 1, 5, 10, 20, 30, 50, 80, 100, 140 and 200 bar and 298 K. The dashed lines mark the amount of oxygen adsorbed in an empty tank taken from the National Institute of Standards and Technology (NIST), 1 bar: 0.9 cm^3^(STP)/cm^3^; 5 bar: 4.6 cm^3^(STP)/cm^3^; 10 bar: 9.2 cm^3^(STP)/cm^3^; 20 bar: 18.5 cm^3^(STP)/cm^3^; 30 bar: 27.9 cm^3^(STP)/cm^3^; 50 bar: 47.1 cm^3^(STP)/cm^3^; 80 bar: 76.4 cm^3^(STP)/cm^3^; 100 bar: 96.2 cm^3^(STP)/cm^3^; 140 bar: 135.8 cm^3^(STP)/cm^3^ and 200 bar: 193.4 cm^3^(STP)/cm^3^). Each point in the graphs represents a different structure. The data points are color coded by void fraction.

**Supplementary Figure 6.** Relationship between absolute gravimetric oxygen adsorption and the largest cavity diameter (LCD) for 2,932 MOF structures at 1, 5, 10, 20, 30, 50, 80, 100, 140 and 200 bar and 298 K. Each point in the graphs represents a different structure. The data points are color coded by void fraction.

**Supplementary Figure 7.** Structure-property relationships between oxygen deliverable capacity, largest cavity diameter (LCD) and void fraction for 2,932 MOF structures at different pressures and 298 K. **a-d.** volumetric and **e-h.** gravimetric oxygen deliverable capacities at 30, 80, 140 and 200 bar storage pressures. The release pressure is kept fixed at 5 bar for all storage pressures. Each point in the graph represents a different structure. The data points are color coded by void fraction (V_f_).

**Supplementary Figure 8.** Relationship between the oxygen deliverable capacity (at 140 bar storage and 5 bar release pressures) and absolute oxygen uptake at 140 bar for 2,932 MOF structures at 298 K. **a.**Gravimetric and, **b.** volumetric adsorption; each point in the graphs represents a different structure. The data points are color coded by void fraction (V_f_).

**Supplementary Figure 9.** Relationship between the oxygen uptake and void fraction at 140 bar for 2,932 MOF structures at 298 K. The data points are color coded and sized by heat of adsorption and the largest cavity diameter, respectively. Each point in the graph represents a different structure.

**Supplementary Table 2.** Geometrical property comparison between 1^st^ quartile (Q1), 3^rd^ quartile (Q3), and the interquartile range (IQR) for the top 1% of structures as a function of storage pressure.

|  | **LCD (Å)** | | | | **Surface Area (m^2^/g)** | | | | **Void fraction (-)** | | | | **Density (g/cm^3^)** | | | |
| --- | --- | --- | --- | --- | --- | --- | --- | --- | --- | --- | --- | --- | --- | --- | --- | --- |
| **Pressure (bar)** | **30** | **80** | **140** | **200** | **30** | **80** | **140** | **200** | **30** | **80** | **140** | **200** | **30** | **80** | **140** | **200** |
| Q1 | 7.01 | 8.17 | 9.08 | 9.79 | 1508 | 2534 | 2965 | 3486 | 0.51 | 0.66 | 0.76 | 0.78 | 0.8 | 0.71 | 0.59 | 0.59 |
| Q3 | 8.32 | 10.88 | 11.95 | 14.83 | 2613 | 3306 | 3720 | 3747 | 0.68 | 0.77 | 0.79 | 0.82 | 0.95 | 0.81 | 0.73 | 0.65 |
| IQR | 1.31 | 2.71 | 2.87 | 5.04 | 1105 | 772 | 755 | 261 | 0.17 | 0.11 | 0.03 | 0.04 | 0.15 | 0.1 | 0.14 | 0.06 |

**Supplementary Note 1**

**GCMC simulations of oxygen adsorption in UMCM-152**

**Supplementary Figure 10.** O_2_ heat of adsorption vs. loading for UMCM-152 calculated from GCMC simulations at 298 K.

Simulation snapshots revealed that at low loading (5 bar), oxygen molecules sit at the framework windows before filling the large UMCM-152 pores at higher pressures (Supplementary Fig. 11).

**Supplementary Figure 11.** GCMC simulation snapshots for O_2_ adsorption in UMCM-152 at different loadings. O_2_ molecules and frameworks atoms are shown in green vdW and CPK representation, respectively.

**Supplementary Method 2**

**Synthesis of UMCM-152**

**Organic linker:**

**Supplementary Figure 12.** Synthetic scheme of ligand **4**.

Dimethyl 5′-bromo-[1,1′:3′,1′′-terphenyl] 4,4′′-dicarboxylate (**1**) was synthesized according to the literature procedure^7^ and the recorded NMR spectrum is in accordance with the reported value. The boronate ester derivative 2 was also synthesized according to the reported procedure.^7^ The syntheses of **3** and **4** were performed by a modified literature procedure.

**Synthesis of 3:**

Compounds **1** (0.8 g, 1.88 mmol) and **2** (0.903 g, 2.82 mmol) were combined in a pressure vessel in 10.4 ml of 1,4-dioxane and deoxygenated with N_2_ for 45 minutes. In a separate flask, K_3_PO_4_ (1.2 g, 5.64 mmol) was dissolved in 1.05 ml of deionized water and purged with N_2_ gas for 1 h. The catalyst Pd(PPh_3_)_4_ (0.054 g, 0.047 mmol) was added to the pressure vessel under positive N_2_ pressure, followed by aq. K_3_PO_4_ solution. The reaction mixture was further purged with N_2_ for 15 minutes and heated at 100 °C for 48 h. After the consumption of **1** (confirmed from thin layer chromatography), the reaction mixture was cooled down and the solvents were evaporated under reduced pressure. Water was added to the residue and the aqueous layer was extracted with dichloromethane three times. The organic layer was collected and further washed with brine solution, filtered and evaporated to obtain the crude product. Further recrystallization with dichloromethane/ acetone solvent mixture gave 0.40 g of product as white solid in 39% yield. ^1^H NMR (500 MHz, CDCl_3_): *δ* 8.71 (t, *J* = 1.5 Hz, 1H), 8.54 (d, *J* = 1.6 Hz, 2H), 8.16 (d, *J* = 8.5 Hz, 3H), 7.87 (s, 3H), 7.77 (d, *J* = 8.5 Hz, 3H), 3.98 (s, 6H) and 3.95 (s, 6H).

**Synthesis of 4:**

The compound **3** (0.37 g, 0.687 mmol) was dissolved in a solution of 1M KOH in 20 ml of 1,4-dioxane/water (2:1) and heated to reflux for 24 hour. The solvents were evaporated under vacuum and the crude product was dissolved in water followed by acidification with concentrated HCl until no further precipitation was observed. The white precipitate was centrifuged and washed with copious amount of water. It was further purified by recrystallization from dimethylformamide/acetone solvent mixture and the product was obtained in 45% yield (0.150 g). ^1^H NMR (500 MHz, DMSO): *δ* 13.2 (bs, 4H), 8.52 (m, 2H), 8.48 (m, 1H), 8.06 (m, 1H), 8.03 (m, 10H).

**NMR spectra**

**
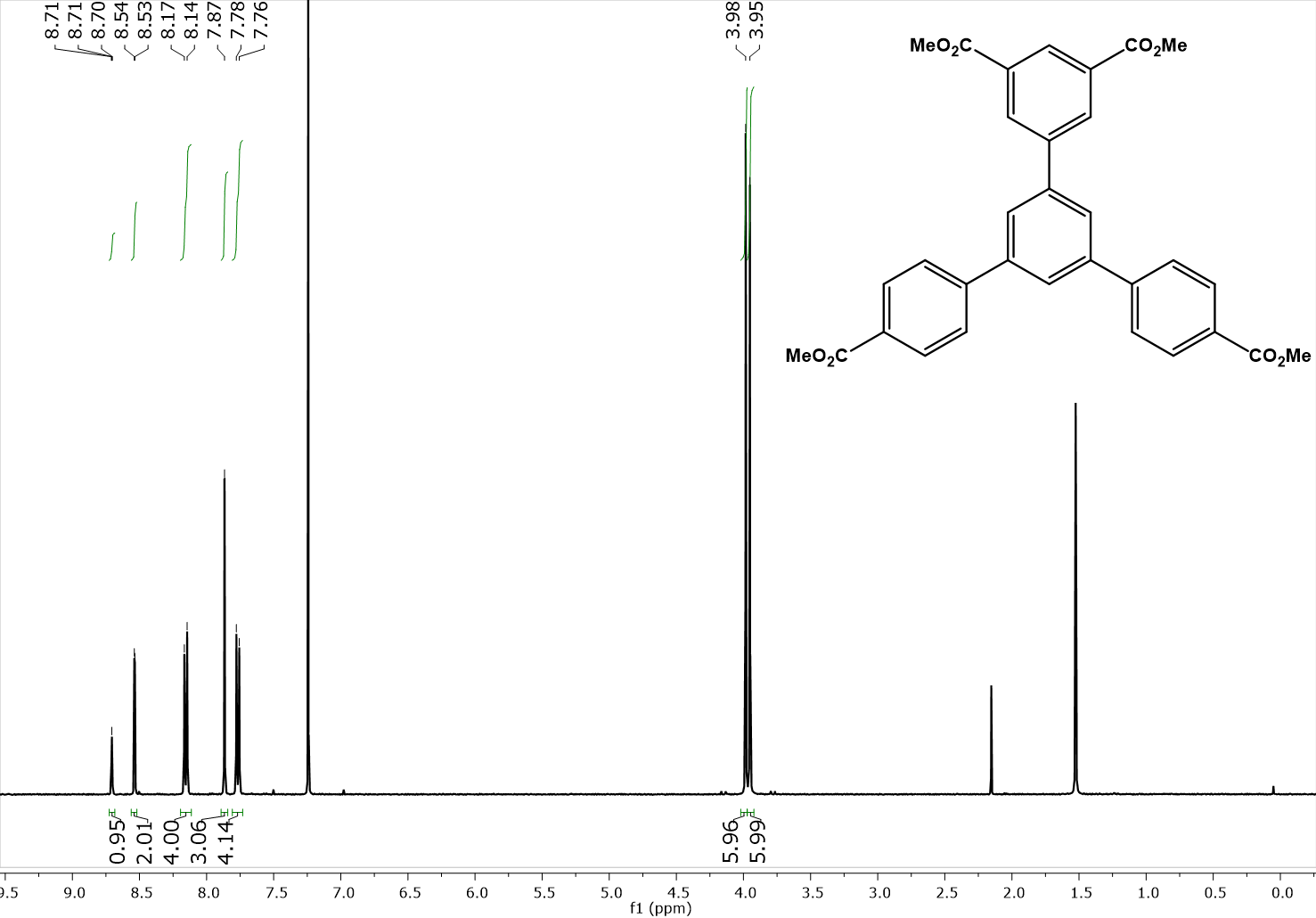
**

**Supplementary Figure 13.** ^1^H NMR spectrum of compound **3** in CDCl_3_.

**
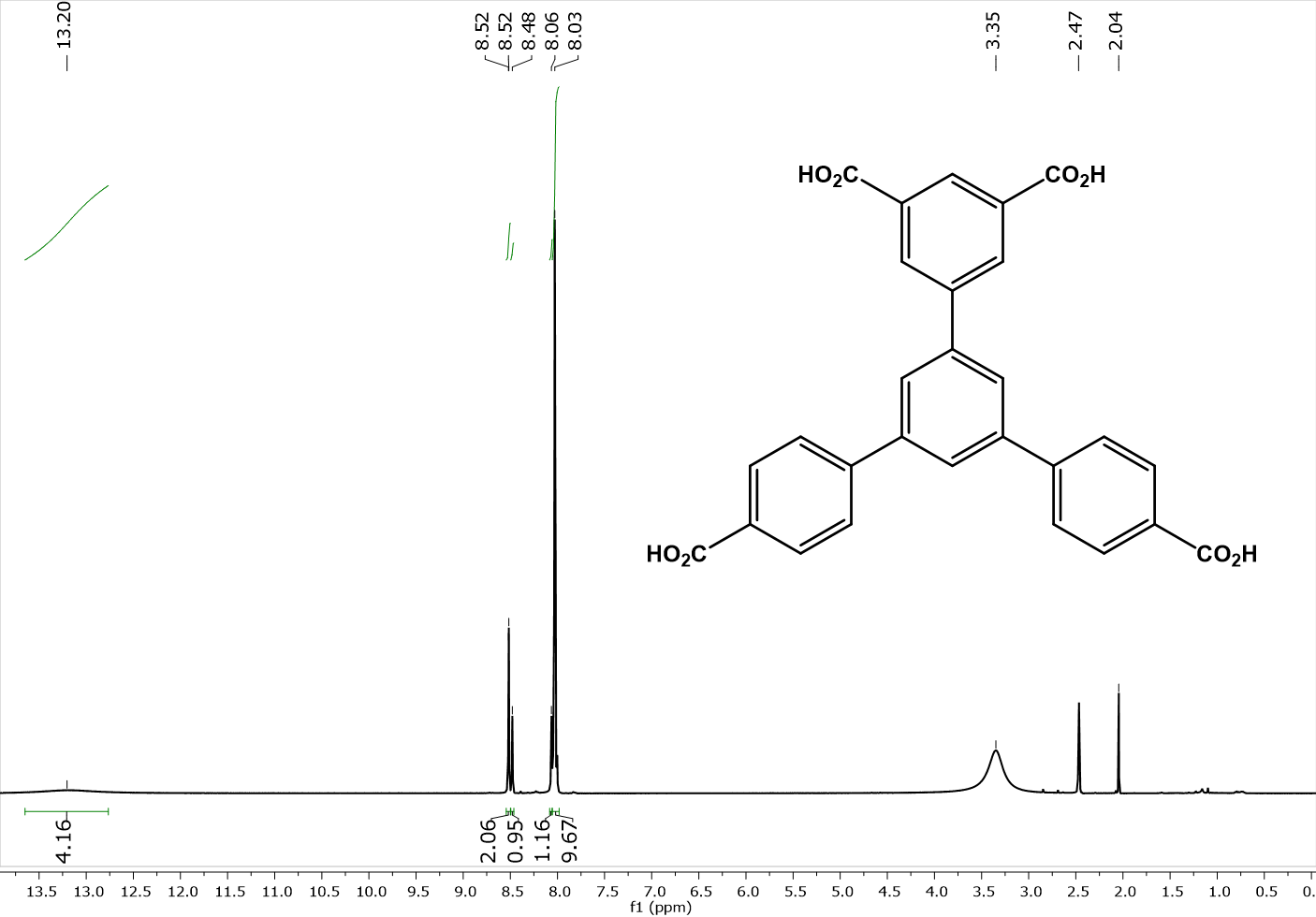
**

**Supplementary Figure 14.** ^1^H NMR spectrum of compound **4** in DMSO.

**Synthesis and activation of UMCM-152:** UMCM-152 was synthesized according to literature^8^ with some modifications. In a 8 dram vial, 50 mg of linker **4** (0.10 mmol) was dissolved in DMF/dioxane/H_2_O (4:1:1, 10 mL) mixture and added 50 µL of 1 M aq. HCl solution. To this mixture, 96 mg of Cu(NO_3_)_2_·2.5H_2_O (0.41 mmol) was added and the contents were sonicated until dissolved. The resulting solution was divided into 10 of 1.5 dram vials equally and heated to 85 °C. (Allowing the UMCM-152 crystals to grow more than 2 hours resulted in the formation of significant amounts of light blue color impurities.) After 2 hours the mother solution was pipetted out and dark blue colored crystals were washed with fresh DMF three times. In some vials minimal amount of fluffy light blue colored impurities were observed, which can easily be removed by a glass pipet after adding fresh DMF. After washing with DMF, the crystals were washed with absolute ethanol three times and soaked in absolute ethanol for 18 hours. Ethanol was removed by supercritical CO_2_ activation and then the sample was transferred into the surface area analyzer tube in glove box filled with argon. Prior to the N_2_ and O_2_ isotherm collection, dark purple UMCM-152 crystals were activated under vacuum at 60 °C for 2 hours and finally at 100 °C for 16 h in order to remove any trace amounts of moisture that could be adsorbed during the sample transfer.

**Supplementary References**

1. Dubbeldam D., Calero S., Ellis D. E., Snurr R. Q. RASPA: molecular simulation software for adsorption and diffusion in flexible nanoporous materials. *Molecular Simulation* **42**, 81-101 (2016).

2. Nazarian D., Camp J. S., Sholl D. S. A Comprehensive Set of High-Quality Point Charges for Simulations of Metal–Organic Frameworks. *Chemistry of Materials* **28**, 785-793 (2016).

3. Rappe A. K., Casewit C. J., Colwell K. S., Goddard W. A., Skiff W. M. UFF, a full periodic table force field for molecular mechanics and molecular dynamics simulations. *Journal of the American Chemical Society* **114**, 10024-10035 (1992).

4. Mayo S. L., Olafson B. D., Goddard W. A. DREIDING: a generic force field for molecular simulations. *The Journal of Physical Chemistry* **94**, 8897-8909 (1990).

5. Zhang L., Siepmann J. I. Direct calculation of Henry’s law constants from Gibbs ensemble Monte Carlo simulations: nitrogen, oxygen, carbon dioxide and methane in ethanol. *Theoretical Chemistry Accounts* **115**, 391-397 (2006).

6. Potoff J. J., Siepmann J. I. Vapor–liquid equilibria of mixtures containing alkanes, carbon dioxide, and nitrogen. *AIChE Journal* **47**, 1676-1682 (2001).

7. Peng Y., Krungleviciute V., Eryazici I., Hupp J. T., Farha O. K., Yildirim T. Methane Storage in Metal–Organic Frameworks: Current Records, Surprise Findings, and Challenges. *J. Am. Chem. Soc.* **135**, 11887-11894 (2013).

8. Schnobrich J. K., Lebel O., Cychosz K. A., Dailly A., Wong-Foy A. G., Matzger A. J. Linker-Directed Vertex Desymmetrization for the Production of Coordination Polymers with High Porosity. *J. Am. Chem. Soc.* **132**, 13941-13948 (2010).
