# Supplementary material for: Computer-aided discovery of a metal–organic framework with superior oxygen uptake
Source: Nat Commun. 2018 Apr 11;9:1378. doi: 10.1038/s41467-018-03892-8 (PMC5895810; doi:10.1038/s41467-018-03892-8)
Supplement: Supplementary file 3 — Description of Additional Supplementary Files [file 41467_2018_3892_MOESM3_ESM.pdf]

Description of Additional Supplementary Files

File Name: Supplementary Movie 1
Description: Evolution of structure-property relationships for oxygen storage in MOFs.
